# Fertility-sparing treatment for patients with endometrial cancer: a bibliometric analysis from 2000 to 2024

**DOI:** 10.3389/fonc.2025.1567806

**Published:** 2025-04-28

**Authors:** Yue Gao, Huali Wang, Meng Jiang, Yumeng Cui, Xiaochuan Yu

**Affiliations:** Department of Gynecology, Dalian Women and Children’s Medical Center (Group), Dalian, Liaoning, China

**Keywords:** fertility-sparing treatment, bibliometric analysis, progestin, molecular classification, endometrial cancer

## Abstract

**Introduction:**

The rising global incidence of endometrial cancer (EC), particularly among younger patients, has established fertility-sparing treatment as a critical focus in gynecological and reproductive medicine. Despite its clinical significance, comprehensive bibliometric analyses in this field remain limited.

**Methods:**

This study conducted the most extensive bibliometric analysis to date, encompassing 506 publications on fertility-sparing EC treatments published between January 1, 2000, and December 22, 2024. Utilizing CiteSpace, JepaC, and VOSviewer, we systematically evaluated contributions across regions, institutions, journals, authors, and keywords to identify emerging research trends.

**Results:**

China and the United States emerged as leading contributors, collectively accounting for 44.3% of publications. Fudan University and Cha University were identified as the most active institutions. Author Chen Xiaojun demonstrated the highest publication output, while Seok J and Seong SJ ranked as the most frequently cited researchers. Keyword analysis revealed five dominant research themes: "progestin," "reproductive outcomes," "age," "prognostic factors," and "myometrial invasion."

**Discussion:**

This analysis delineates the evolving landscape of fertility-sparing EC research, highlighting persistent emphasis on hormonal therapies and prognostic determinants. The findings underscore the need for standardized treatment protocols and long-term fertility outcome studies. By mapping research trajectories and visualizing knowledge networks, this study provides actionable insights to guide future investigations in reproductive oncology.

## 1 Introduction

The incidence of EC has increased by 132% over the past 30 years. Although studies report an increasing trend in all age groups, the number of cases in women under 40 years has doubled, accounting for 4.2% of the low-grade EC patient population in the United States (1). Younger patients with EC (aged 40 years or younger) typically present with endometrioid, focal, hyperdifferentiated tumors confined to the endometrium or superficial myometrium, which are associated with a good prognosis. Approximately 79.1% of young patients are diagnosed with the histopathologic type of Endometrioid carcinoma, 72%–80% present with stage IA disease, and more than half have highly differentiated tumors. The 5-year disease-specific survival rate for this group is 93%–96% (2–5). The tumor’s low malignancy and favorable prognosis make fertility-preserving treatment a viable option for young patients with EC.

Currently, most domestic and international guidelines and consensus outline specific indications for conservation therapy in patients with EC. These include the following criteria: the tumor is a highly differentiated endometrioid carcinoma; it is confined to the endometrium without evidence of extrauterine metastasis; there are no contraindications to drug therapy or pregnancy; and the patient wishes to preserve fertility and is well-informed about the treatment options (6). In 2023, the International Federation of Gynecology and Obstetrics (FIGO) introduced a revised staging system for EC (7), defining stage IA1 as a noninvasive histologic type limited to polyps or confined to the endometrium. Additionally, the European Society of Gynecological Oncology (ESGO), the European Society for Radiotherapy and Oncology (ESTRO), and the European Society of Pathology (ESP) have collectively issued 48 evidence-based recommendations for conservative therapy (8). These guidelines address factors such as age limits, health status, obesity, Lynch syndrome, estrogen-progestin receptor status, and molecular typing.

Ongoing research aims to improve complete tumor remission rates and pregnancy outcomes for patients undergoing fertility-preserving therapy for EC. Achieving these goals holds substantial significance, both in terms of advancing treatment for malignancy and enhancing fertility preservation.

Bibliometrics is a scientific research method that uses quantitative analysis of literature data to reveal trends and patterns in scientific research. It objectively evaluates the contributions of academic groups and individual researchers by examining authorship, country affiliations, journals, citations, and publication dates of selected articles. This method enables the identification of trends in specific fields and the ranking of academic groups and individuals (9). By analyzing literature citations, collaborative networks, and keyword patterns, bibliometrics highlights key contributors, research hotspots, and future directions in a research area (10). In this study, we used bibliometric analysis to review the literature on fertility-sparing treatments for EC published over the last two decades. Our objective was to characterize the research landscape and predict future trends and key areas of investigation. Specifically, we analyzed publication patterns, author and institutional contributions, journal impact, and the evolution of research themes and keywords. This study provides a comprehensive perspective on research dynamics in the field of fertility-sparing treatments for EC.

## 2 Materials and methods

### 2.1 Searching strategy

The Web of Science Core Collection (WoSCC) was selected for this study due to its superior accuracy in document type annotation compared to databases like Scopus, Medline, and PubMed. With coverage of over 12,000 academic journals, WoSCC is widely recognized as a gold standard for bibliometric analysis. On December 22, 2024, we conducted a systematic search for articles published between January 1, 2000, and December 22, 2024, using the following formula: ((TS=(“Endometrial Neoplasm*”) OR TS=(“Endometrial Carcinoma*”) OR TS=(“Endometrial Cancer*”) OR TS=(“Endometrium Cancer*”) OR TS=(“Endometrium Carcinoma*”) OR TS=(“Endometrial neoplasia”) OR TS=(“Endometrial tumo*”) OR TS=(“Endometrium neoplasm”) OR TS=(“Endometrium tumour”) OR TS=(“Eeoplastic endometri*”))) AND ((TS=(“Fertility preserv*”) OR TS=(“Fertility sparing”) OR TS=(“Conservative treatment”) OR TS=(“Conservative Surgery”) OR TS=(“Conservative management”))).

### 2.2 Inclusion criteria

The literature screening for this study was based on the following inclusion criteria:

1. Full-text publications related to fertility-sparing treatment for EC
2. Articles and review manuscripts written in English
3. Articles published within the defined timeframe (January 1, 2000, to December 22, 2024).

### 2.3 Analytical tools

To comprehensively analyze research trends in fertility-sparing treatments for endometrial cancer, we employed the following bibliometric tools for data processing and visualization: VOSviewer 1.6.20 was utilized to visualize co-authorship networks, keyword clusters, and citation relationships, with metrics including collaboration density, keyword co-occurrence frequency, and citation impact to identify international/institutional collaboration patterns, high-frequency keywords, and assess the academic influence of highly cited literature. CiteSpace 6.3.R1 conducted co-citation analysis to detect influential authors/journals and research hotspots via metrics such as keyword burst detection (e.g., “molecular classification” as a recent trend) and co-citation network clustering, enabling tracking of research theme evolution from basic research to clinical guideline integration. Pajek 5.19 performed advanced network partitioning and sensitivity analysis of co-authorship networks using metrics like network modularity and node centrality to resolve hierarchical institutional collaboration structures and validate the stability of high-output author groups.

### 2.4 Exclusion criteria

**Figure 1:**
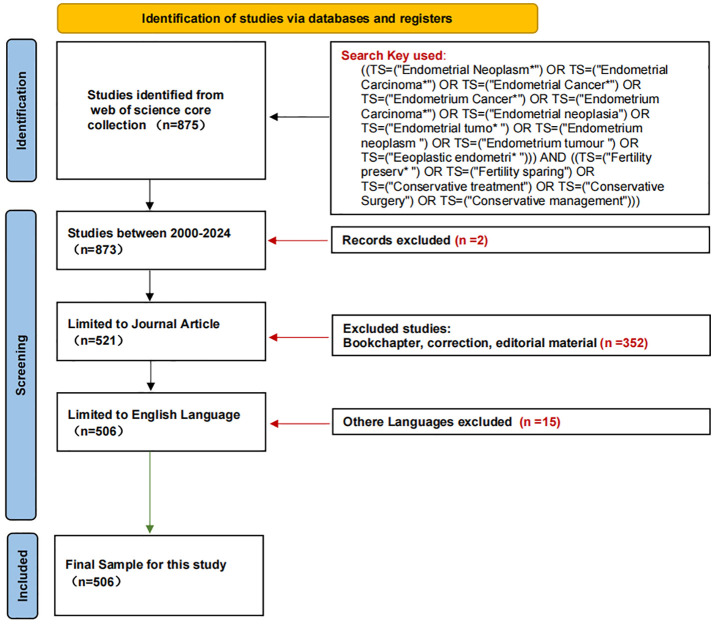
Flow chart.

Topics not related to fertility-sparing treatment for EC, non-English language articles, and publications that were conference abstracts, news, briefings, etc. were excluded. The plain text version of the papers was exported for analysis. **Figure 1** shows the literature flow chart. This study primarily utilized the Web of Science Core Collection (WoSCC) as the main source; however, this approach might have excluded relevant studies published in other databases such as PubMed or Scopus. To address this limitation, a supplementary search was conducted using the same combination of keywords in both PubMed and Scopus, resulting in the addition of 38 new articles. Consequently, **Figure 1** has been updated to reflect a total of 544 articles.

### 2.4 Data analysis

We used VOSviewer1.6.20, Pajek5.19 and CiteSpace6.3.R1 software to analyze the details of authors, sources, titles, keywords, cited references, etc. of the articles. VOSviewer is a scientific mapping tool developed by Prof. Van Eck and Prof. Waltman from the Center for Scientific and Technological Research of the University of Leiden, and is used to visual bibliometric analysis, mainly for analyzing details such as co-authors, countries and keywords (11, 12).

CiteSpace was developed by Prof. Chaomei Chen of Drexel University in 2004. It can be used to analyze and measure the co-occurrence frequency of key information (keywords, authors, regions, and citations) in an article and present the trend of related research (13). We used VOSviewer version 1.6.20 to collect information on the place of origin of the studies, keywords used in the studies, total number of papers, quantity and quality of cited literature, collaboration between researchers affiliated with different institutions, and clustering of keywords in the studies. The results derived from VOSviewer were further processed using Pajek 5.19 to obtain more sensitive and comprehensive results. CiteSpace 6.3.R1 was used to understand the keyword occurrence patterns and geographic time zones of published articles. The use of these software applications ensured that research and trends in EC fertility preservation therapy were analyzed from all perspectives.

## 3 Result

### 3.1 Overview of publication status

**Figure 2:**
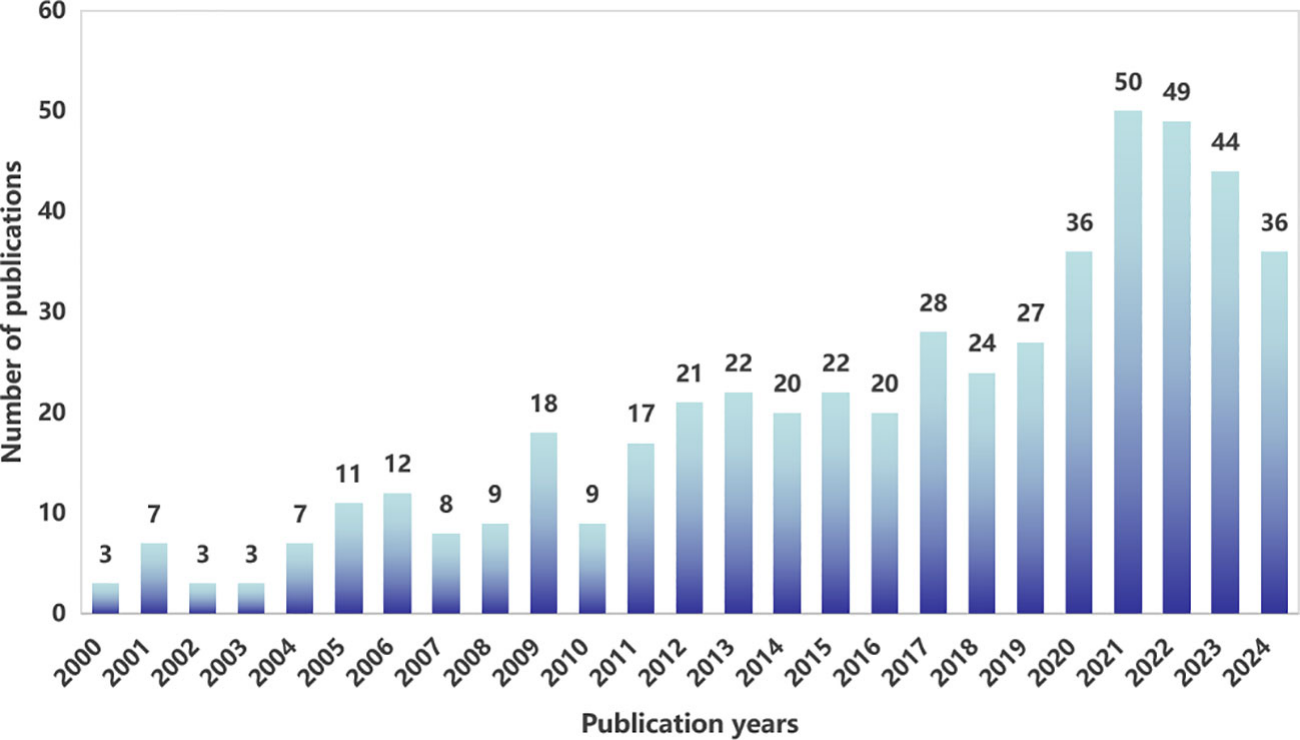
Annual publication volume chart.

From 2000 to 2024, WoSCC tabulated a total of 506articles on the topic of fertility-sparing treatment for EC therapy, and the annual publication distribution is shown in **Figure 2**. We can find that the number of articles published in the related studies is generally on the rise, and the annual publication in 2021 is at 50 articles, the highest value in recent years.

### 3.2 Analysis of the number of national publications

**Figure 3:**
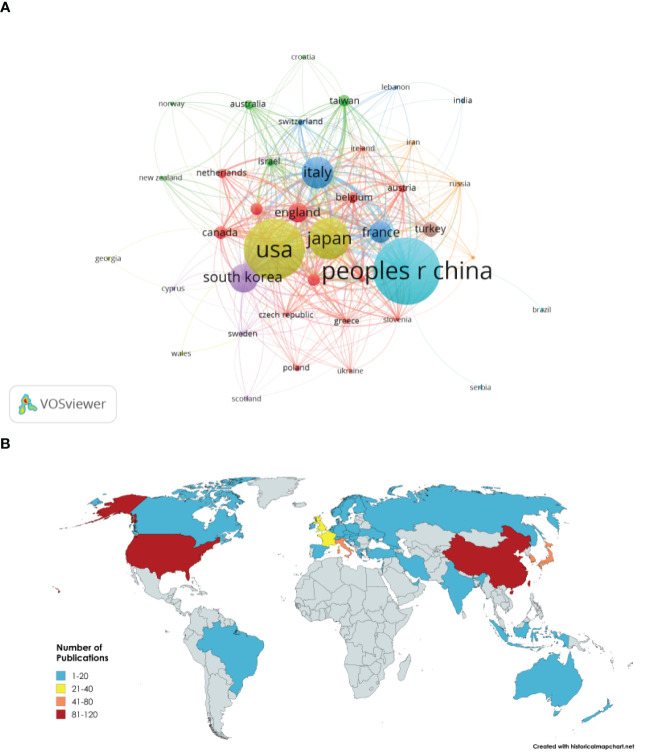
**(A)** Network visualization of countries and regions. **(B)** Visualization of global publication volume.

Research on the application of fertility-sparing treatment for EC has been conducted in 44 countries and regions (**Figure 3A**). The leading five countries in this field are China, the United States, Japan, Italy, and South Korea. China accounts for 23.12% of the total publication volume. Among the top five countries/regions in terms of paper publication, Italy ranks first with a citation-to-publication ratio of 51.77 (2485/48), indicating that the overall quality of its published papers is generally high. On the world map, the number of articles published by each country/region is labeled and presented as in **Figure 3B**.

### 3.3 Analysis of institutional publications

**Figure 4:**
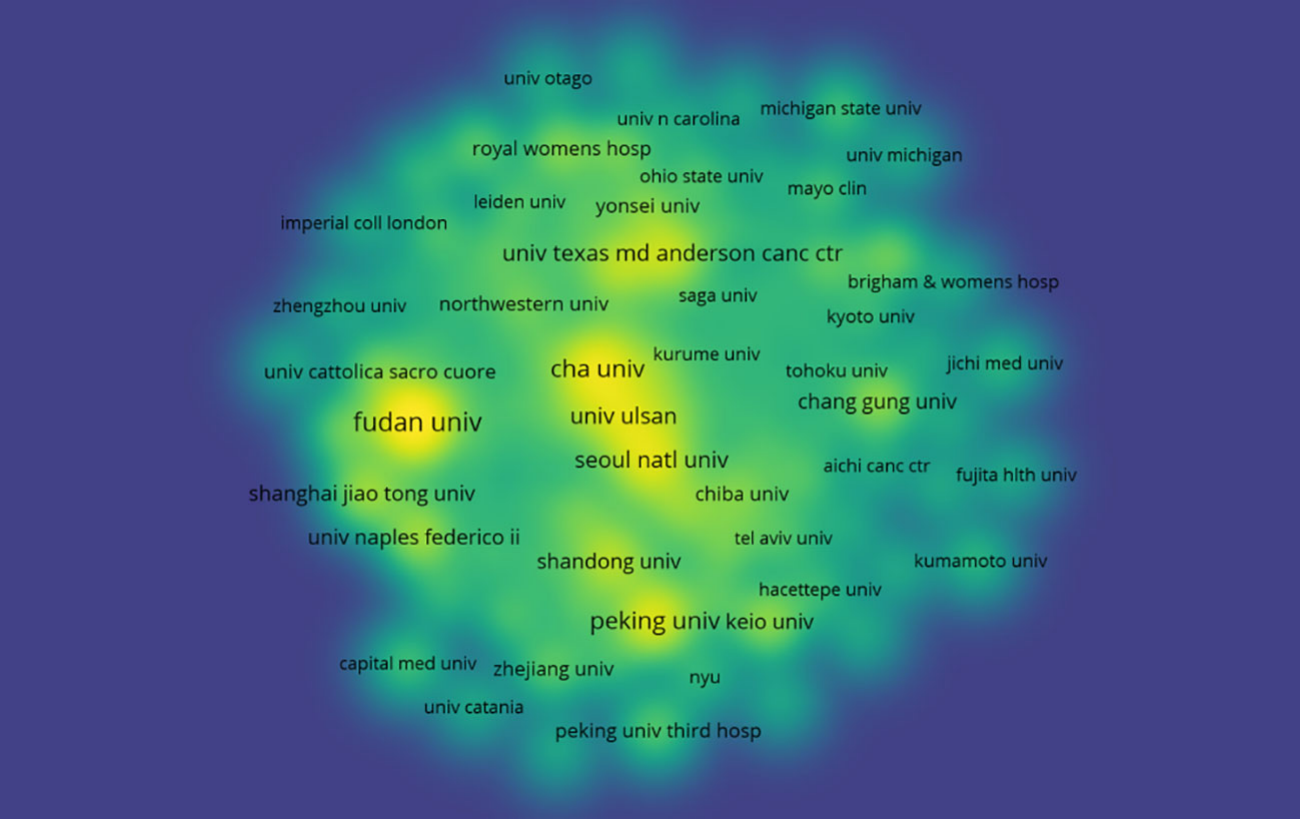
Heat map of institutions.

To explore the contributions of institutions to fertility-sparing treatment for EC, we analyzed the number of publications from each institution. A total of 756 institutions worldwide has systematically published articles on fertility-sparing treatment for EC. The Fudan University published the most papers (26 papers, 315 citations, 12.12 citations per paper). Cha University ranked second (17 papers, 694 citations, 40.82 citations per paper), followed by the Peking University (16 papers, 133 citations, 8.31 citations per paper) (**Figure 4**). Further analysis revealed that both domestic and foreign institutions tend to collaborate with units within their own country. Therefore, we advocate for strengthening cooperation between domestic and foreign institutions to break down academic barriers.

### 3.4 Analysis of publication volume and journal impact

**Figure 5:**
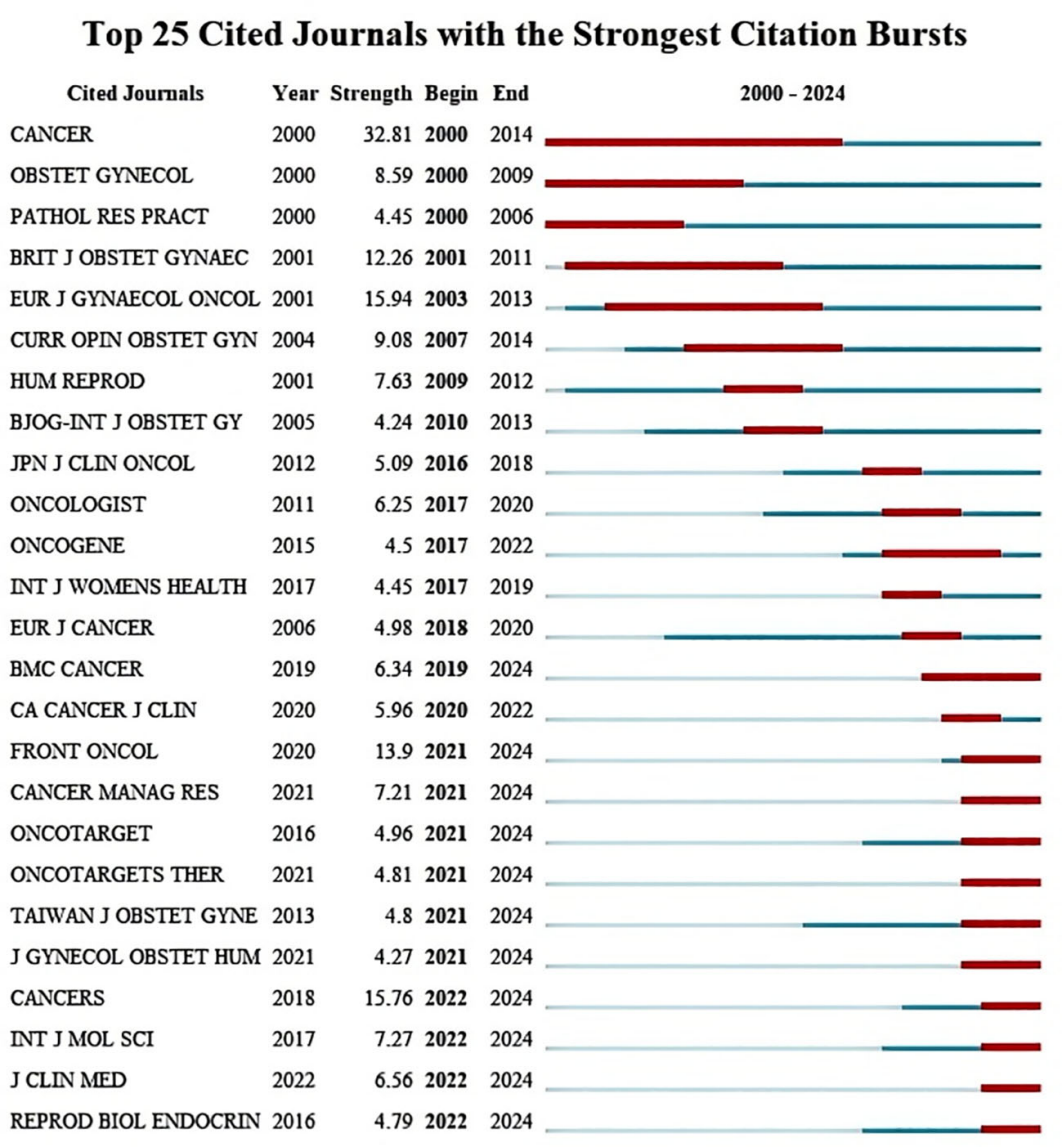
Journal pop-up map.

**Figure 6:**
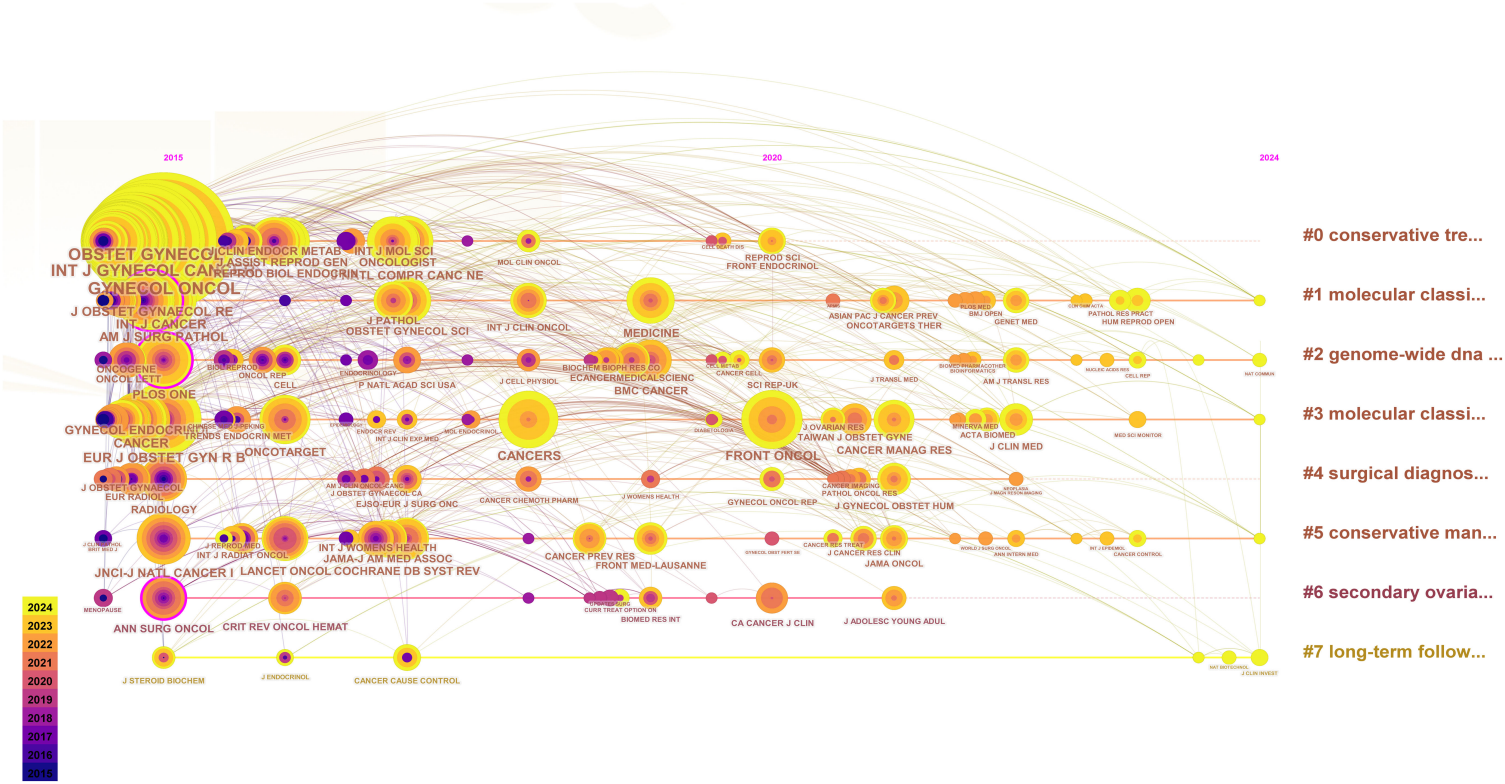
Journal time zone map.

We counted the top 5 most frequently cited journals, GYNECOL ONCOL (449 times), INT J GYNECOL CANCER (352 times), OBSTET GYNECOL (352 times), AM J OBSTET GYNECOL (291 times), and J CLIN ONCOL (245 times). The top 25 journals with the strongest bursts, sorted by year, are displayed through the mutation mapping of the cited journals. The intensity and duration of increase in the number of citations for each journal in a given period is indicated by the emergence intensity histogram, where a higher emergence intensity indicates a significant increase in the journal’s influence in the field (**Figure 5**). The evolution of cited journals under different research-based themes is further reflected by plotting the timeline mapping from 2015-2024, which gives a more holistic response to the course of the research and its trends (**Figure 6**).

**Figure 7:**
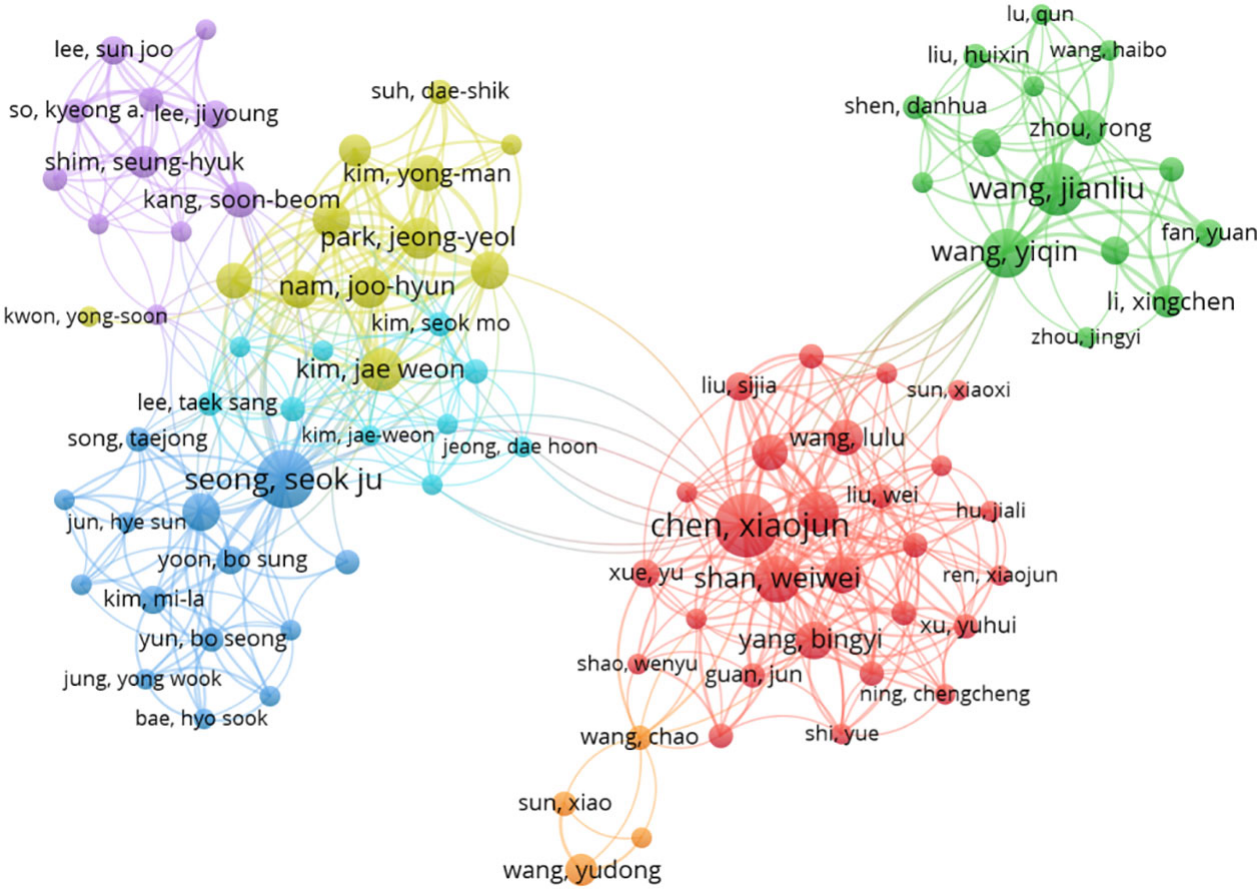
Collaborative network of co-authors.

The top 5 authors with the largest number of publications in this research area are: Chen Xiaojun (19 publications, Obstetrics and Gynecology Hospital of Fudan University, Shanghai, China.) Seong, Seok Ju (16 publications, Department of Obstetrics and Gynecology, CHA Gangnam Medical Center, CHA University, Seoul, Korea.) Wang Jianliu (13 articles, Department of Obstetrics and Gynecology, Peking University People’s Hospital, Beijing, China.) Wang Yiqin (11 articles, Department of Obstetrics and Gynecology, Peking University People’s Hospital, Beijing, China.) Koskas, Martin (11 articles, Division of Gynecologic Oncology, Bichat University Hospital, Paris, France.). The top 5 cited authors were Seong, Seok Ju (673 citations, average citation rate: 42.06), Nam, Joo-Hyun (534 citations, average citation rate: 66.75) Kim, Jae Weon (492 citations, average citation rate: 54.67), Kim, Tae-Jin (490 citations, average citation rate: 81.67), Park, Jeong-Yeol (481, average citation rate: 60.13). Indicating that their research holds a high reputation and influence. the network between authors is visualized using CiteSpace (**Figure 7**).

### 3.5 Keyword analysis

**Figure 8:**
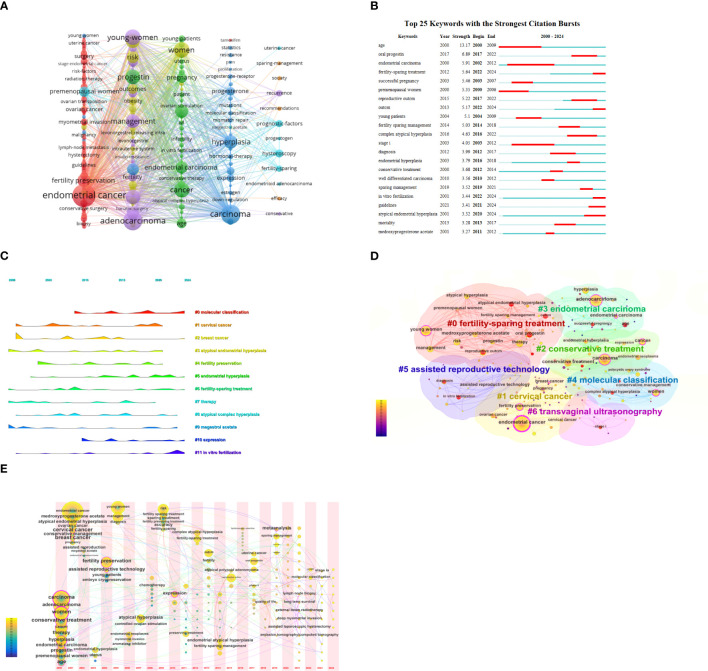
**(A)** Network visualization of keywords. **(B)** Keyword pop-up map. **(C)** Keyword clustering diagram. **(D)** Keywords time zone map. **(E)** Keyword clustering volcano map.

By analyzing keywords, we can quickly understand the state and development direction of a field. VOS viewer and CiteSpace were used to draw different visual clustering maps of keywords used in the published articles. The clustering network visualization of keywords were created on VOS viewer (**Figure 8A**). Excluding synonyms for “EC”, “fertility protection” and redundant terms such as “female”, we found that the top keywords used were progestin, reproductive outcomes, age, prognostic-factors, myometrial invasion. CiteSpace software was used to complete the analysis of the appearance of keywords used in the studies of metabolic dysfunction in fertility-sparing treatment for EC (**Figure 8B**). Using the co-citation relationship of the literature on the keywords of the article clustering, and the formation of the corresponding set of clusters, the size and color of the clusters show the development of a cluster of the history and development of the scale, presenting a certain area of the hotter research topics and research areas. (**Figure 8C**). We used CiteSpace to create volcano map and time zone map visually display the changes in research hotspots over time (**Figures 8D, E**).

## 4 Discussion

Scientometric analysis software facilitates the systemic evaluation of scientific literature. It enables the examination of a comprehensive body of research on a specific topic, efficiently extracting and summarizing relevant information through quantitative methods. This approach provides an intuitive overview of data, highlighting trends such as whether a scientific topic has experienced heightened research interest during a particular period, and offers insights to predict future developments in the field. Key metrics analyzed include the number of publications and citations as well as the impact of publications across dimensions such as country, institution, author, journal, scientific category, and keyword engagement.

### 4.1 General information study

A scientometric analysis of fertility-sparing treatment for EC revealed a consistent upward trend in both. This indicates that the topic is likely to remain a focal point of research over the next 5 years. The United States and China emerged as leading contributors to this field. Studies conducted in the U.S. and China have contributed to updates in clinical guidelines, including the 2023 FIGO staging system, which integrates molecular typing into risk assessment to facilitate personalized treatment decisions. Notably, the United States has produced the most highly cited papers and established the most international collaborations, indicating a prominent role in advancing research on fertility-sparing treatment for EC.

### 4.2 Progress in the study of indications for fertility-sparing treatment for EC

In the Keyword pop-up map and Keyword clustering diagram, terms such as “age,” “well-differentiated carcinoma,” “myometrial invasion,” “stage IA,” and “molecular classification” are closely associated with the indications for fertility-sparing treatment in EC. These findings align with the study’s exploration of such indications throughout. Current national and international guidelines and consensus for fertility-sparing treatment in EC specify the following criteria: highly differentiated endometrioid carcinoma (G1 grade) confined to the endometrium, no evidence of extrauterine metastases, no contraindications to medication or pregnancy, and a well-informed patient’s desire for fertility preservation (6). Before initiating conservative therapy, professional multidisciplinary counseling is strongly recommended, especially for patients with genetic syndromes (8, 14, 15). The Chinese expert consensus provides additional requirements: (1) Patients aged ≤40 years with a strong desire for fertility (2) Positive expression of both estrogen receptors (ERs) and progesterone receptors (PRs). (3) Normal serum CA125 levels. (4) Molecular typing for non-specific molecular subtype (NSMP) (16).

The efficacy of conservative therapy for patients with G2 grade EC and shallow myometrial infiltration remains inconclusive. In 2023, the European Society of Gynecologic Oncology (ESGO/ESHRE/ESGE) emphasized the importance of individualized treatment regimens for patients with moderately differentiated (G2) EC (8). A retrospective study conducted by the Gynecologic Collaborative Group on Malignant Neoplasms (GCIG) analyzed 23 patients with G2 EC, reporting a complete remission (CR) rate of 74%, a median time to CR of 6 months, a median follow-up of 35 months, a recurrence rate of 41%, and a pregnancy rate of 30% (17). These results align with findings from Park et al. (18), which indicated that the CR rate of progestogen therapy for G2 EC is comparable to that in G1 EC. However, achieving CR typically takes longer, and there is an increased risk of pathological progression during treatment, underscoring the need for careful monitoring and further research. The potential for fertility preservation in patients with EC and myometrial infiltration remains a subject of debate. A retrospective cohort study reported a lymph node metastasis rate of 0.5% in G1 EC patients without myometrial infiltration, which increased to 1.6% in patients with up to 50% myometrial infiltration (19). Similarly, a multicenter study involving 23 patients with EC and superficial myometrial infiltration reported a CR rate of 73.9% (17/23) and a recurrence rate of 47.1% (8/17) following oral progestogen therapy (18). Fertilization sparing treatment for non-muscle invasive endometrial cancer has recently been reported in a single center (20). Due to the high recurrence rate and the limited availability of robust data, fertility-sparing treatment in patients with myometrial infiltration should be approached cautiously. A comprehensive assessment, incorporating advanced imaging and reproductive medicine consultation, is essential to inform individualized treatment decisions.

The time zone and peak plots from the keyword analysis (**Figures 8D, E**) indicate a gradual increase in research on the molecular typing of EC in recent years. The fifth edition of the WHO Classification of Tumors of the Female Genital Organs (21) and the National Comprehensive Cancer Network Guidelines for Tumors of the Body of the Uterus (6) recommend molecular typing as a critical component in the management of EC. The proposed molecular classification is based on the ProMisE method, which categorizes EC into four subtypes: POLE mutation (POLE mut), Microsatellite instability/mismatch repair-deficient (MSI/MMR-D), p53 abnormality (p53 abn), and NSMP type. The POLE mut subtype accounts for approximately 6%–8% of EC cases, mainly involves endometrioid carcinoma, is prevalent among young women, and is associated with a good prognosis, making it suitable for conservative treatment. Conversely, patients with the MSI/MMR-D subtype show poor responses to progesterone therapy, have a high recurrence rate following conservative treatment, and experience unfavorable long-term prognosis; however, they may benefit from immunotherapy (22–24). The p53abn subtype is hormone therapy-resistant and poses a high risk of treatment failure under conservative treatment. The NSMP subtype is the most common in nursery-treated patients with endometrial cancer, comprising 70%–80% of patients undergoing conservative treatment. These patients demonstrate higher complete remission rates and lower recurrence rates compared to the MSI/MMR-D and p53 abn subtypes (25). L1CAM, a transmembrane protein involved in cell migration and adhesion, has emerged as an independent risk factor for poor prognosis in NSMP-type EC. It is associated with TP53 mutations, high tumor grade, and lymphovascular interstitial infiltration (26). However, the role in the success of conservative therapy for patients with NSMP subtypes requires further investigation. In addition, it is essential to integrate the identified research trends with clinical applications. Molecular classifications, such as ProMisE subtypes, play a pivotal role in guiding personalized treatment decisions. For instance, patients with tumors harboring POLE mutations tend to be younger and exhibit a relatively favorable prognosis, making them ideal candidates for fertility-sparing therapy. In relevant studies, progestogen therapy has demonstrated a high rate of complete remission in these patients and may thus represent a more suitable option. Conversely, for patients with MSI/MMR-D tumors, given their potential resistance to progestin, alternative treatment strategies, such as immunotherapy, should be explored to prevent recurrence and improve outcomes. By incorporating molecular triage into clinical practice, we can enhance the precision of treatment plans, thereby improving both therapeutic efficacy and patient outcomes.

### 4.3 Advances in fertility-sparing treatment for EC methods

Progestin-based medications combined with hysteroscopic treatment are among the most effective treatments for preserving fertility in patients with EC. Hysteroscopy in combination with progesterone therapy demonstrated significantly superior outcomes compared to single therapy in terms of lesion clearance rate and pregnancy outcomes (p<0.05). However, it is crucial to strictly control the uterine cavity pressure at less than 70 mmHg to minimize the risk of tumor dissemination. We have systematically compiled disease prognosis outcomes associated with distinct therapeutic approaches (**Table 1**), with comprehensive analyses of treatment modalities to be elaborated in subsequent sections.

**Table 1:** Prognosis of different fertility-sparing treatments for EC.

Treatment type	Procedure /Drug	Disease Stage	Complete Remission Rate	Recurrence Rate	Key Reference
Hysteroscopic Surgery+Progestin	Three-step resection+LNG-IUS or MPA	IA (G1/G2)	89.3% (28/31)	7.7% (2/26)	Falcone et al., 2017 (38)
Progestin Monotherapy	Oral MPA (250-500 mg/d) or MA (160-320 mg/d)	IA (G1)	76.3% (95% CI: 70.7-81.1%)	30.7% (95% CI: 21.0-42.4%)	Zhao et al., 2007 (51)
Hysteroscopic Cold Knife	Lesion excision + hormonal therapy	IA (G1)	78.6% (11/14)	18.2% (2/11)	Giampaolino et al., 2019 (37)
LNG-IUS + GnRH-a	Intrauterine system + gonadotropin-releasing hormone agonist	IA (G1) with contraindications to oral therapy	68.5% (13/19)	22.1% (3/13)	ESGO Guidelines, 2023

#### 4.3.1 Surgical treatment in fertility-sparing treatment for EC

In 1846, diagnostic curettage (D&C) became the “gold standard” for diagnosing and treating intrauterine lesions. While the use of ultrasound guidance has enhanced the safety of blind surgical techniques to some extent, it cannot fully replace direct surgical visualization and intervention (27–29). The introduction of diagnostic hysteroscopy in 1980 (30) marked a significant advancement in uterine cavity management. Over time, hysteroscopic technologies have evolved, with reduced diameter instruments enabling minimally invasive procedures. These fine-diameter hysteroscopes allow direct visualization of the uterine cavity and facilitate guided therapeutic interventions. A meta-analysis conducted 23 years ago involving 26346 cases demonstrated hysteroscopy’s high diagnostic performance, reporting an overall sensitivity of 86.4% and specificity of 99.2% (31). Compared to traditional diagnostic curettage, hysteroscopy offers superior diagnostic and therapeutic capabilities, including precise sampling under direct vision and subsequent pathological examination. Consequently, both domestic and international guidelines recommend hysteroscopy-guided endometrial biopsies for the diagnosis and treatment of EC.

The hysteroscopic endometrial biopsy techniques encompass key-clamp biopsy, crocodile clamp biopsy, snake clamp biopsy, and bipolar electrode chip biopsy. Among these methods, grasp biopsy, initially proposed by Bettocchi in 2002 (32), is widely regarded as the most appropriate technique for endometrial biopsy in women of childbearing age. The crocodile forceps, characterized by their dentate jaws, are capable of simultaneously grasping and cutting tissue, thereby enabling the collection of larger tissue samples. During the procedure, the clamp is opened to locate the target area, advanced to obtain a 0.5-1 cm tissue strip (while avoiding contact with the myometrium), closed to secure the specimen, and then removed intact (33, 34). Regarding biopsy coverage, Professor Wang Jian-Liu, who ranks third in terms of publication volume in China, introduced the “5+x” hysteroscopic biopsy method. This approach has been incorporated into the Chinese Expert Consensus on Fertility-Preserving Treatment for Endometrial Cancer. It involves obtaining tissue samples from the anterior, posterior, left, right, fundus, and focal regions of the uterine cavity, which enhances the detection rate of lesions and facilitates a comprehensive, continuous, and accurate assessment of the endometrial response to treatment (35).

Hysteroscopic lesion excision can effectively reduce the tumor burden and improve the efficacy of pharmacological conservative treatment. In 2005, Mazzon (36) first introduced the ‘three steps’ technique for hysteroscopic resection of focal endometrial tumors. This approach involves (1) excision of the tumor tissue, (2) resection of the endometrium surrounding the lesion (4–5 mm lateral to the lesion tissue), and (3) removal of the underlying myometrium (3–4 mm beneath the lesion). The technique has been successfully implemented in clinical practice, including achieving a full-term pregnancy in a patient with stage IA EC following combined progesterone therapy. Giampaolino et al. (37) used the ‘three-step’ hysteroscopic technique in 14 patients with EC. Post-surgical treatment with a levonorgestrel extended-release intrauterine system resulted in a CR rate of 78.6% and a recurrence rate of 18.2% at a 24-month follow-up. Similarly, Falcone et al. (38) reported a CR rate of 89.3%, a recurrence rate of 7.7%, and pregnancy and live birth rates of 93.3% and 86.6%, respectively, among 28 patients with EC treated using a three-step electrodessication method.

Hysteroscopic cold knife surgery, an evolution of traditional hysteroscopic thermal energy instruments, has gained widespread clinical application for managing various uterine diseases in recent years (39, 40). The 2024 Chinese Expert Consensus on Quality Control and Evaluation Standards for Surgical Treatment of Endometrial Cancer recommends the use of cold knives or scissors to individually remove lesions. In contrast, international guidelines do not explicitly endorse the hysteroscopic cold knife technique for this patient population. Existing studies indicate that the hysteroscopic cold-knife technique eliminates the risk of electrical or thermal injury, offers superior protection of the endometrium, and facilitates better evaluation of myometrial infiltration due to the absence of thermal effects (41–43). However, the technique may increase the risk of intraoperative bleeding and water intoxication because it lacks the electrocutaneous closure of small blood vessels. In addition, for patients with myometrial infiltration, it remains unclear whether the local electrocutting of endometrial lesions might aid in initiating local endometrial inactivation and reducing recurrence risk compared to cold knife surgery. Large-scale studies are currently lacking in this area. Future research should evaluate the efficacy and complications of hysteroscopic electrosurgical versus cold-knife resection as fertility-sparing treatments for patients with EC.

Hysteroscopy has become the primary modality for EC diagnosis and monitoring during treatment. However, some scholars are concerned that hysteroscopic surgery or manipulation may lead to the migration of tumor cells through the fallopian tubes into the abdominal cavity. One significant risk factor is the excessive pressure exerted by the fluid used to expand the uterus during hysteroscopic procedures, which could facilitate the mechanical opening of the fallopian tubes, allowing endometrial tumor cells to pass through and migrate. Baker et al. (44) and Levêque et al. (45) conducted studies in which they found that the rate of positive peritoneal cytology results was 37% when dilatation pressures were up to 150 mmHg (1 mmHg = 0.133 kPa), compared to only 1% at pressures below 100 mmHg. Kudela et al. (46) conducted a meta-analysis of data from nine clinical studies involving 1015 patients with endometrial cancer and showed that the risk of tumor cell migration to the peritoneal cavity increased when hysteroscopy was performed with fluid pressures exceeding 100 mmHg. Baker et al. (44) observed a significant decrease in the number of endometrial cells in the abdominal cavity when the pressure during hysteroscopy was less than 70 mmHg. Furthermore, DeSousa et al. (47) used pressures below 80 mm Hg and CO2 as the bulking medium during gas hysteroscopy, performing peritoneal cytology both before and after the procedure. They found no endometrial cells in any of the patient’s post-procedure. While there are no prospective studies to confirm that hysteroscopy increases the spread and clinical progression of tumor cells within the abdominal cavity, it is reasonable to assume that the risk of endometrial tumor cell metastasis does not increase when using lower distending pressures. Therefore, the distending pressure must be strictly controlled during hysteroscopy in patients with EC.

#### 4.3.2 Progesterone therapy in fertility-sparing treatment for EC

Progesterone was first introduced for the treatment of EC by Kistner (48) in 1959, making it the earliest class of endocrine therapeutic agent used clinically for this condition. Currently, oral progestins are the first-line pharmacological option for EC in patients seeking fertility preservation. Recommended regimens include continuous daily oral administration of megestrol acetate (160-320 mg/d) or medroxyprogesterone acetate (250-500 mg/d), with a median time to complete remission of 6 months. Reported rates of complete remission and recurrence are 76.3% (95% CI: 70.7%–81.1%) and 30.7% (95% CI: 21.0%–42.4%), respectively (49). However, oral progestins may cause side effects, such as weight gain and hepatic impairment (50). For patients who are progesterone-resistant/ineffective (SD, PR-negative, relapse) or those unsuitable for progesterone therapy due to factors like obesity, abnormal liver/renal function, hypercoagulable states, thromboembolic lesions, other pharmacological therapies, such as LNG-IUS and GnRH-a, should be considered.

The first limitation of progestin therapy is the development of resistance. While the complete remission rate reaches 70% in patients with early hyperdifferentiated EC, approximately 30% of patients either fail to respond or experience disease progression (26). Zhao et al. (51) successfully established a progesterone-resistant endometrial cancer cell line, revealing that resistance may be attributed to an imbalance of progesterone receptor isoforms, specifically, the downregulation of PR-A mRNA, upregulation of PR-B/PR-A mRNA, and increased expression of PR proteins expression. Additional mechanisms include aberrant expression or persistent activation of epidermal growth factor receptor (EGFR) and its downstream signaling pathways (52). In addition, tumor stem cells have a stronger DNA repair ability than tumor-differentiated cells, enabling adaptation to environmental changes and ensuring timely repair of tumor tissue damage, which fosters drug resistance. In a collaborative study led by Prof. Chen Xiaojun from Fudan University in China and Prof. Ma Ding’s team from Huazhong University of Science and Technology (53), genomics and proteomics analyses were performed on 229 formalin-fixed, paraffin-embedded (FFPE) tissue pairs, including samples from 81 patients under 40 years of age with early emerging endometrial carcinomas (EEEC). This study identified higher chromosomal instability in progesterone-insensitive patients. The interaction between SIGLEC10 and ESR1 proteins, facilitated by SIGLEC10Q144K, was found to promote progesterone resistance, as confirmed through protein interaction analysis and co-immunoprecipitation (Co-IP) assay. In addition, the research conducted by Chen Xiaojun’s team on SIGLEC10 gene mutations and their association with drug resistance offers a promising target for the development of novel progesterone sensitizers. Relevant clinical trials have already been initiated (NCT05897432). Several studies have explored strategies to overcome progesterone resistance, such as the upregulation of PRB by chlorpromazine to increase the sensitivity of progesterone-resistant endometrial cancer cells to progesterone drugs. Additionally, the combination of neural cell adhesion molecules and medroxyprogesterone acetate (MPA) has been found to enhance PRB expression and counteract resistance (54, 55). Resistance has also been linked to methylation of the PR gene, leading to low or absent PRB expression. Wei et al. (56) improved progesterone resistance by reversing the methylation of the PR gene in cancer cells using a demethylating drug (5-aza-2-deoxycytidine). Furthermore, epithelial-mesenchymal transition (EMT), a key process in cancer development, has been associated with progesterone resistance. Zhou et al. (57) revealed that the transcription factor DACH1 regulates EMT by affecting the c-Jun/Notch1/Hes1 signaling pathway to preserve the sensitivity of endometrial cancer cells to progesterone, which further inhibits uterine endometrial cancer development. However, the clinical applications of relevant studies require further exploration. Overall, addressing the mechanism of progesterone resistance and improvement of the regimen will be the focus of future research. This will significantly improve the clinical benefits of progesterone therapy.

Another significant limitation of progestin therapy is the absence of reliable biomarkers for predicting treatment response. Progesterone is a steroid hormone that binds to progesterone receptors (PR) to exert its effects. Currently, the most reliable biomarker for endocrine therapy in EC is PR, with tumors exhibiting high PR expression usually responding better to treatment. Subsequently, high ER and/or AR expression was proposed as a possible benefit of endocrine therapy. However, even when hormone receptor status is positive, EC may exhibit resistance to endocrine therapy, contrary to findings from some studies, in which endocrine therapy remained effective in patients lacking hormone receptor expression (58). Another study involving 14 patients with G1 EC found a significant decrease in ER and PR expression in the immunohistochemistry of endometrial biopsy specimens after progesterone treatment (median H-scores before and after ER treatment: 183 and 104, respectively, p=0.013; PR: 110 and 40, respectively, p<0.001). This indicates a negative feedback loop from PR to ER signaling at the protein level. This study also observed glucocorticoid Receptor (GR) levels were elevated (23 vs. 47, p=0.003), whereas the androgen receptor (AR) exhibited a different response. Neither pre- nor post-treatment changes in steroid receptor (SR) levels were associated with response to progesterone therapy thus neither pre-therapy SR levels nor changes in SR receptor status correlated with clinical response to progesterone therapy, highlighting the complex, context-specific interactions between SR and their ligands (59). Furthermore, the effectiveness of endocrine therapy has been poorly assessed using the hormone receptor status alone. A recent study identified four proteins with preexisting antibodies (EEF1E1, ILVBL, SRPK1, and NUDT5) whose expression was significantly upregulated in progesterone-insensitive EEEC using proteomic analysis, suggesting that they could be used as potential markers for fertility preservation therapy (53). Overall, further research is essential to identify reliable predictive markers for response to conservation therapies. In the future, approaches from multiple perspectives, such as genomics, metabolomics, and proteomics, and the emergence of reliable research results in one of these directions will be of great significance in clinical practice.

#### 4.3.3 Treatment efficacy summary

To enhance clarity and address reviewer feedback, we present a summary table of treatment outcomes for fertility-sparing therapies in EC:

## 5 Limitation

This study has several limitations. First, the analysis was confined to the Web of Science (WoS) Core Collection, which may not comprehensively capture all publications in this field. While WoS is widely regarded for its high-quality indexing, it may underrepresent non-English literature, regional studies from institutions not indexed in its journals, and certain publication types (e.g., conference papers, technical reports). This could introduce bias in collaboration networks, keyword trends, and author/institutional rankings. Although PubMed and Scopus searches have been incorporated, grey literature may still be overlooked. Future efforts should focus on integrating clinical trial registration platforms, such as ClinicalTrials.gov, to enhance the comprehensiveness of data analysis and ensure robust conclusions. Second, the data extraction focused on publications from the past 25 years (2000–2024), potentially missing earlier foundational research that may inform long-term treatment evolution. Finally, although bibliometric analysis was performed objectively using software, the interpretation of results inherently involves researcher subjectivity, particularly in keyword clustering and trend forecasting.

## 6 Conclusion

Despite limitations, this study represents the first bibliometric analysis of fertility-sparing EC treatment trends over 25 years, developing a knowledge map encompassing annual publication volume, country/institutional collaborations, source journals, and keyword clusters to guide researchers in journal selection, collaboration opportunities, and hotspot identification; these insights highlight the critical role of molecular classification (e.g., ProMisE subtypes) in treatment optimization and underscore the need for standardized guidelines incorporating multi-omics data. We recommend future research focus on multi-center trials evaluating long-term outcomes of fertility-sparing protocols, integration of real-world evidence for prognostic models, and development of precision therapies targeting EC molecular subtypes.

## Data Availability

The datasets presented in this study can be found in online repositories. The names of the repository/repositories and accession number(s) can be found in the article/supplementary material.
